# Real-Time fMRI Neurofeedback with War Veterans with Chronic PTSD: A Feasibility Study

**DOI:** 10.3389/fpsyt.2016.00111

**Published:** 2016-06-21

**Authors:** Mattia I. Gerin, Harlan Fichtenholtz, Alicia Roy, Christopher J. Walsh, John H. Krystal, Steven Southwick, Michelle Hampson

**Affiliations:** ^1^Yale Child Study Center, Yale School of Medicine, New Haven, CT, USA; ^2^Division of Psychology and Language Sciences, University College London (UCL), London, UK; ^3^Anna Freud Centre, London, UK; ^4^Department of Radiology and Biomedical Imaging, Yale School of Medicine, New Haven, CT, USA; ^5^Department of Veteran Affairs, National Center for PTSD, West Haven, CT, USA; ^6^Department of Psychiatry, Yale School of Medicine, New Haven, CT, USA; ^7^Bennington College, Bennington, VT, USA

**Keywords:** PTSD, war veterans, neurofeedback, real-time fMRI, resting-state, functional connectivity

## Abstract

Many patients with post-traumatic stress disorder (PTSD), especially war veterans, do not respond to available treatments. Here, we describe a novel neurofeedback (NF) intervention using real-time functional magnetic resonance imaging for treating and studying PTSD. The intervention involves training participants to control amygdala activity after exposure to personalized trauma scripts. Three combat veterans with chronic PTSD participated in this feasibility study. All three participants tolerated well the NF training. Moreover, two participants, despite the chronicity of their symptoms, showed clinically meaningful improvements, while one participant showed a smaller symptom reduction. Examination of changes in resting-state functional connectivity patterns revealed a normalization of brain connectivity consistent with clinical improvement. These preliminary results support feasibility of this novel intervention for PTSD and indicate that larger, well-controlled studies of efficacy are warranted.

## Introduction

Post-traumatic stress disorder (PTSD) is among the most impairing and common of psychiatric conditions, with a lifetime prevalence of 8–10% and a 12-month prevalence rate between 3.7 and 4.7% (1, 2). Yet, extant data indicate that about 30–50% of patients do not respond to current evidence-based psychological therapies or pharmacological interventions, and some subgroups (such as combat-exposed patients) show even higher rates of treatment-resistance and drop-out [(3, 4); Institute of Medicine Report]. Over the last few decades, despite the dramatic growth in the literature of the neurobiological underpinnings of PTSD, very few neuroscientific discoveries have been translated into effective and novel treatments (5). Currently, the most effective interventions have been informed by psychological theories and behavioral data (such as cognitive behavioral therapy (CBT) and other trauma-based therapies) (5, 6). Pharmacological interventions for PTSD, such as selective serotonin reuptake inhibitors (SSRIs), which were introduced due to their antidepressant effects, have demonstrated modest treatment response (5). To date, only two medications, both selective SSRIs, have FDA approval for the treatment of PTSD. In civilian treatment seeking populations, fewer than half of patients achieve full remission on SSRIs. The rates of non-response or partial response to these medications among combat-exposed military, particularly those with chronic PTSD are comparable or worse to those of the civilian patient population (7–11).

Therefore, it is paramount that we begin to bridge the gap between neurobiological findings of PTSD and clinical practice. The development of neuroscientifically informed treatments has the potential to complement and enhance current interventions, thus increasing treatment effectiveness and reducing treatment-resistance and drop-out rates (12, 13).

A few studies have investigated the potential of electroencephalography (EEG) neurofeedback (NF) as a neuroscientifically informed intervention for PTSD (14–16). Although EEG NF has important features, it has the limitation that targeting specific brain areas, such as the amygdala, is difficult if not impossible with EEG. As our understanding of the brain circuits involved in PTSD and other disorders advances, there is increasing demand for a NF technique that can more directly leverage neuroscientific knowledge by allowing us to target specific brain areas. Real-time fMRI neurofeedback (rt-fMRI NF) presents this opportunity.

Real-time fMRI neurofeedback is a relatively new technique that allows us to target localized brain areas (or to entrain specific spatial patterns of brain activity), providing a wonderful opportunity for developing neuroscience-guided, targeted interventions. In rt-fMRI NF, participants receive contingent visual (or auditory) feedback about a specific aspect of their brain activity pattern, and practice trying to control that aspect of their brain function using feedback as a training signal (17, 18). Many studies have shown that rt-fMRI NF training can be used to teach participants to exert volitional control over their own neurophysiological response (18–21). In a variety of subject populations, participants have used rt-fMRI NF to successfully regulate activity in the amygdala, anterior cingulate, and insula, among other brain regions and circuits (19, 21–23). As participants learn to modulate their brain response, it has been shown that behavioral and neurophysiological changes occur that are specific to the brain region or network targeted during the NF (21, 24–30). Due to the ability of NF to modify cognition, behavior, affect, and neurophysiology, the potential applications of rt-fMRI NF are being investigated in clinical treatment settings (31, 32). Current literature suggests that rt-fMRI NF may be able to reduce symptom levels and also normalize brain activity across diverse psychiatric and neurological conditions (21, 27, 28, 33–41).

To the best of our knowledge, this pilot study represents the first attempt to test the viability of rt-fMRI NF for PTSD. Three Operation Enduring Freedom (OEF), Operation Iraqi Freedom (OIF), and Operation New Dawn (OND) veterans with chronic PTSD (i.e., more than 3 months) were recruited and underwent three rt-fMRI NF training sessions (of about 30 min each). A functionally localized region of the amygdala was targeted during NF due to (i) its well-established involvement in PTSD pathophysiology, hyperarousal, and anxiety (42–45) and (ii) because extant rt-fMRI NF data suggest that subjects can learn to modulate its activity level (23, 41). In particular, participants were trained to modulate their amygdala activation during a symptom provocation paradigm (i.e., participants listened to audio-recorded narratives of their own traumatic memories). By training participants to control emotion-related brain activity patterns that are activated during trauma recall, the intervention directly targets a possible neural correlate of their symptomatology. In addition to clinical symptom assessment before and after the NF, functional neuroimaging data pre- and post-NF training were also collected. Although any clinical intervention should ultimately demonstrate effectiveness by reducing symptoms and/or increasing well-being, incorporating neurophysiological data as an outcome variable in clinical trials is recommended as a means of better elucidating treatment response (12, 46, 47).

The overarching aim of this pilot research project was to investigate the clinical feasibility of rt-fMRI NF with a highly vulnerable patient population with chronic PTSD. There were three main hypotheses. First, it was hypothesized that all participants would tolerate the intervention well. This was expected because (i) neuroimaging studies have used symptom provocation paradigms with PTSD patients (48, 49), (ii) various studies using other NF techniques (e.g., EEG NF) showed that NF is feasible with PTSD patients (14–16), and (iii) studies of rt-fMRI NF have been successfully performed with other patient populations that show disturbances in affect regulation and cognition (37, 38, 41, 50). Second, in line with other clinical NF studies, it was hypothesized that, post-intervention, patients would show a reduction in symptoms (15, 27, 28, 31, 37, 38, 50). Third, it was hypothesized that normalization in resting-state functional connectivity (rsFC) of the amygdala would occur (51–57). In particular, rsFC changes were expected to be consistent with those found in other PTSD resting state and intervention studies that used neuroimaging data as an outcome measure (58). In particular, we expected to find (i) an increase in rsFC between the amygdalae and regulatory medial and orbitofrontal areas, and (ii) a decrease in rsFC between the amygdalae and limbic regions and salience network areas.

Importantly, if these hypotheses would be satisfied, only the feasibility, but not the efficacy, of this treatment would be established. At this stage, any post-intervention changes in functional connectivity or symptoms cannot be attributed directly to the NF. For such causal inference to be made a larger sample and the presence of a control/placebo group would be necessary.

## Materials and Methods

### Design

This pilot study investigated the feasibility of rt-fMRI NF for treating PTSD patients. A short-term longitudinal design was adopted in order to measure symptoms and rsFC changes before and after the NF intervention. No control group was used for this unblinded pilot intervention.

### Participants

Three OIF/OEF/OND veterans with chronic PTSD were recruited through the Veterans Affairs (VA) Connecticut Healthcare System, in West Haven, CT, USA. The three participants will be referred to as participant A, B, and C. Participants received compensation for their time and travel expenses. The study was performed in agreement with a research protocol approved by the Human Research Protection Program at Yale University and the Human Subjects Subcommittee at the VA Connecticut Healthcare System. Subjects provided written consent at both institutions.

Entry criteria included (i) a formal diagnosis of PTSD, as confirmed by the Clinician Administered PTSD Scale (CAPS) and with a total severity score of ≥50; (ii) chronic PTSD symptoms for at least 1 year; (iii) no concurrent Axis I disorders at the time of assessment, with the exception of non-threshold mood and anxiety symptoms; (iv) concurrent psychotropic treatments only if participants were on stable doses for at least 3 months; (v) no new behavioral treatments initiated for the past 3 months; and (vi) meeting the standard safety requirements for MR scanning.

### Measures

#### Behavioral Measures

##### Demographics

Clinical records regarding current and past PTSD-related treatments were available through the VA Connecticut Healthcare electronic medical record system. Patient demographic information was also collected, including age, handedness, gender, ethnicity, education, and employment status.

##### Structured Interviews

Post-traumatic stress disorder symptoms were assessed (past-month and life time) using the CAPS for DSM-IV-revised 1998 (59). The CAPS was administered by experienced raters who demonstrated excellent inter-rater reliability (i.e., Kappa coefficients were between 0.80 and 0.90 for all interviewers). The Structured Clinical Interview for DSM IV-TR (SCID) was also administered to assess for comorbid Axis I disorders (60).

##### Questionnaires

The Combat Exposure Scale (CES) (61) was administered to assess combat exposure. (i) The Beck Depression Inventory, version II (BDI-II) (62), (ii) the military version of the PTSD checklist (PCL-M) for DSM-IV (63), and (iii) the State-Trait Anxiety Inventory (STAI) (64) were used to assess, respectively, depression, PTSD, and anxiety symptoms.

##### Trauma Scripts

A similar procedure to that described by Lanius et al. (65) and Pitman et al. (66) was used to generate the trauma imagery scripts. Briefly, in collaboration with each participant, script narratives from their six most traumatic life events were created and ranked from most to least traumatic. Two different scripts were formulated for each trauma memory (i.e., different wordings and descriptions). The scripts were rich in imagery, narrative accounts as well as descriptions of the emotional and physiological states experienced during the traumatic event (e.g., sweaty hands, tense muscles, etc.). The scripts were recorded by the same male voice across all participants. The recordings were then edited so that each single trauma script would last exactly 60 s. Then, six audio recordings of 286 s were produced and played back to the participants during the NF runs. Each NF recording contained 26 s of silence, 60 s of trauma script, then 70 s of silence, then 60 s of a different trauma script (of similar traumatic intensity), and again 70 s of silence.

#### Magnetic Resonance Imaging Data Acquisition Protocol

All magnetic resonance imaging (MRI) data acquisition was done using a 1.5-T Siemens Sonata scanner (Siemens Medical Systems, Erlangen, Germany). When imaging regions with high susceptibility, improvements in signal with increasing scanner strength are offset by increases in the susceptibility artifacts. In previous work, we optimized a NF protocol on our 1.5-T scanner for training people to control signal from high susceptibility regions of the brain (67). As the amygdala is also a region of susceptibility, we used the same scanner and sequence in this study.

##### Structural Image Acquisition

Every scanning session started with a structural localizer scan. During the first scanning session, the structural localizer was followed by a high-resolution sagittal scan, collected using a magnetization prepared rapid gradient echo (MPRAGE) sequence (TR = 2400 ms; TE = 3.54 ms; TI = 1000 ms; flip angle = 8°; matrix size = 192 × 192; FoV = 240 mm^2^; voxel size = 1.3 mm × 1.3 mm × 1.2 mm; Bandwidth = 180). This was used to register data to the Colin brain, thereby transforming it into the Montreal Neurological Institute (MNI) coordinate system (68). On the following scanning sessions instead of the high-resolution structural image (MPRAGE), a short lower-resolution sagittal T1-weighted scan was collected. This sagittal scan is required for slice alignment.

Then a T1-weighted spin echo axial-oblique anatomical scan (i.e., conventional anatomical scan) was collected with 31 contiguous, 3.1 mm-thick AC-PC aligned axial-oblique slices with coverage extending up from the bottom of the cerebrum (TR = 537 ms; TE = 11 ms; flip angle = 90°; matrix size = 256 × 256; FoV = 200 mm^2^; voxel size = 0.8 mm × 0.8 mm × 3.1 mm; bandwidth 130). This structural image is necessary for registrations of the functional data into higher resolution space (i.e., MPRAGE image) and for the registration of the target and control regions (used during the NF).

##### Functional Imaging

The collection of structural images was followed by the acquisition of functional data at the same slice locations as the axial-oblique T1-weighted data. All functional images were acquired using a T2*-sensitive gradient-recalled single shot echo-planar pulse sequence (TR = 2000 ms; TE = 30 ms; flip angle = 80°; matrix size = 64 × 64; FoV = 200 mm^2^; 3.1 mm × 3.1 mm × 3.1 mm; interleaved acquisition; bandwidth = 2604). The collection of functional images began, during every scanning session, with a short functional run from which a single volume was extracted to be used as the functional reference volume. A longer, but otherwise identical, functional data acquisition protocol (143 volumes, 286 s) was used also for all the other functional data acquisitions, including (i) two resting-state runs collected during every scanning session (i.e., two resting runs of 4 min and 46 s each), (ii) the functional localizer run used to select the region of interest (ROI) to be targeted during the NF sessions, and (iii) the real-time NF runs.

#### Image Data Processing

Images were motion corrected using SPM (http://www.fil.ion.ucl.ac.uk/spm/). Except where noted, all other analyses (i.e., real-time processing, t-maps for functional localizers, and seed-connectivity maps) were conducted using Yale BioImage Suite software package (69). All t-maps were smoothed using a 6-mm full-width at half maximum Gaussian kernel. All registrations were visually inspected.

#### Real-Time fMRI Data Acquisition Protocol

All subjects underwent three separate NF scanning sessions in which the target ROIs within the amygdala were functionally defined. The functionally defined ROIs were based on functional localizers (i.e., a trauma audio-script and frightening movie scenes) during the first scanning session for each subject.

##### Regions of Interest

For a detailed description of functional ROI processing, see the procedure described by Hampson et al. (67). Briefly, after the first scanning session, the data from the functional localizer were analyzed using the general linear model (GLM) to identify the 30 most active voxels within the amygdalae (with a cluster threshold of four voxels) to anxiety provoking stimuli (sometimes a scary movie was used and sometimes a personal trauma). The selection of the 30 voxels was done with a customized MATLAB program before the first NF session. At the start of each NF session, the ROI was transformed from the functional space in which it was defined (that is, the functional space of the first day when the functional localizer was collected) into the anatomical space of that same day via a rigid registration with nearest neighbor interpolation.

A control region, which included all the white matter, was defined by transforming the white matter from the template MNI brain into the patient’s same anatomical space used during the target ROI registration. The white matter is chosen as a control region during the NF to control for global drift and arousal levels. The real-time analysis program used these two regions during the NF sessions. To adjust for global signal fluctuations in the real-time data, we followed the approach introduced by deCharms et al. (21) and plotted the percent signal change from the running mean of the target region minus percent signal change from the running mean of the control region.

##### Real-Time Pre-Processing

After the anatomical images were collected, the target ROI and the white matter control region were translated into the functional space of the current scanning day via a concatenation of two rigid registrations. First, the anatomical space of the first day was mapped to the anatomical space of the current day. Then, the anatomical space of the current day was mapped to the space of the functional reference scan of the current day. Once the ROI and the control region were registered into the functional space of the current scanning session, the real-time NF could begin.

##### Real-Time fMRI System

The rt-fMRI system that provides visual feedback during the NF scans is described extensively in two previous published studies (27, 28, 67).

### Procedure

#### Timeline

Prior to the NF intervention, subjects completed all questionnaires, the SCID and the CAPS were administered, and the trauma scripts were created.

The CAPS clinical interview was conducted between one to 3 weeks prior to and after the NF training. Within 1 week before and after the NF training, the baseline and post-intervention resting-state scans were collected. On the day of these resting-state scans, before entering the MRI scanner, participants completed the PCL-M, the BDI-II, and the STAI. The NF sessions took place every 2–4 days (so the sequence of three sessions lasted 7–9 days depending on the participant). On each NF training day, before entering the scanner, participants also completed the PCL-M, BDI, and STAI.

#### Scanner Procedure

During each scanning day, participants completed the PCL-M, BDI-II, and STAI questionnaires before entering the scanner (note that the BDI and STAI scores will be presented only in the online Supplementary Materials document – in Figures S1 and S2 in Supplementary Material). Once in the scanner, the functional image acquisition began with the resting-state runs. The light was dimmed in the scanner room and subjects were required to rest for about 10 min with their eyes open (~5 min per resting run). On the first day, the resting-state runs were followed by the functional localizer runs (i.e., used to create the target ROI). During functional localizer runs, participants watched movie clips with frightening scenes or listened to a trauma audio-script for the activation blocks and rested for the baseline periods. Participant B also had two NF runs on this day (targeting the right amygdala defined anatomically).

**Figure 1:**
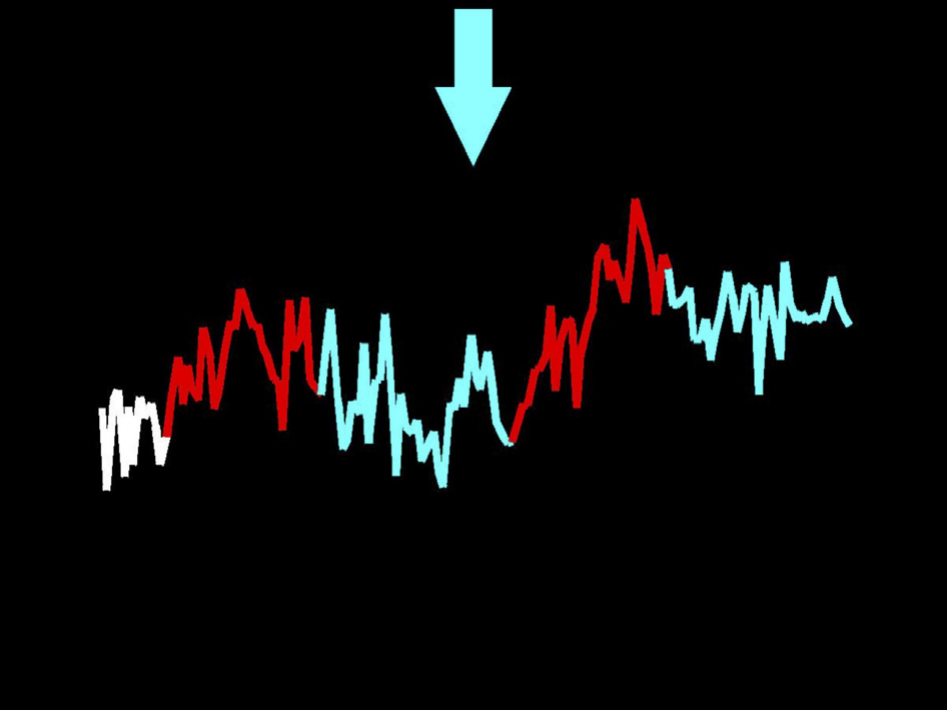
**Screen capture of the display a participant viewed taken at the end of a neurofeedback run**. The participant heard personalized trauma scripts during the red/increase periods, and silence during the blue/decrease and white/rest periods. Their task was to bring the line back down in the blue blocks.

After the first scan, all participants completed three NF scanning days where they received NF from a functionally defined region of the amygdala (bilaterally or unilaterally, depending on where the 30 most active voxels were during the functional localizer scan). Participants received detailed instructions before entering the scanner for the first NF session and received another brief reminder before the first NF run during each NF scanning day. Briefly, participants were informed that the line on the screen (Figure 1) represented the real-time activity (with about 4–6 s delay) of their own amygdala, an area of the brain where activity tends to increase when one is anxious. Participants were also told that an arrow at the top of the screen would cue them as to their current task throughout the run (Figure 1). A white arrow pointing forward would indicate a rest period (no task), a red arrow pointing up would indicate they should listen to the script and allow their amygdala activity to increase, and a blue arrow pointing down would cue them to try to decrease activity in their amygdala (Figure 1). The feedback was provided in the form of a line graph at the center of the screen. The line graph showing them their brain activity pattern was color-coded to match the arrows, so the line segments drawn during the rest block were white, those drawn during the increase/provocation block were red, and those drawn during the decrease block were blue (Figure 1). It was emphasized to the patients that they should bring the blue line down or at least stop it from increasing.

During each NF run, a 286-s audio file was played to patients (see Trauma Scripts for description of these files) while they viewed NF on the display screen. First, participants rested for 26 s at the very beginning (this, as explained above, was indicated by the white arrow). Then, as they listened to the first trauma audio-scripts (60 s), they were cued to allow their amygdala activity to increase (shown by the red arrow pointing upwards). When the trauma audio-script stopped, the participants were cued to decrease activity for 70 s (indicated by a blue arrow pointing down, as shown in Figure 1). Then, a second 60-s trauma script was played during which they could allow their amygdala to increase, and this was followed by the last period of attempted reduction of amygdala activity (70 s). Participants were allowed and encouraged to use any strategy to help them decrease activity. Participants completed five to six NF runs (of 286 s each) during each NF session (i.e., about 30 min in total). Trauma scripts were ranked from least traumatic to most traumatic. Participants began each NF session with runs in which they only heard the less traumatic scripts. How quickly each participant progressed to more traumatic scripts was determined by the patient. This self-paced approach was designed to maximize the comfort and sense of control experienced by the participant. However, all participants were able to use, at least on the last two NF scanning days, the most traumatic audio-scripts.

After completing all of the NF sessions, subjects returned for one final scanning session in which only resting-state functional data were collected, in order to allow comparison of resting-state data before and after NF.

### Data Analysis

#### Behavioral Data

Due to the small sample size, no formal statistical or group analysis was performed on questionnaires and clinical interview data. Rather each subject was used as their own control for the analysis. In particular, symptom changes were calculated for each individual and compared to standardized threshold guidelines for clinical and statistical significance.

#### Offline Analysis of Resting-State Functional Connectivity Data

In addition to motion correction (see Image Data Processing), pre-processing involved regressing from the data time-course signals of no interest, including (i) cerebrospinal fluid and white-matter signals, (ii) subject motion parameters, and (iii) temporal drift, as well as low-pass filtering (<0.1 Hz). Resting-state data were analyzed using a seed-based FC approach. Specifically, a ROI-to-whole-brain connectivity analysis was performed. The ROI was created by transforming the bilateral amygdalae from the MNI brain into the subject functional space. This anatomically defined ROI was used rather than the functionally defined NF target region as the latter varied across individuals. Seed-connectivity maps were computed for the resting runs of each scanning session for each participant. These maps indicate how synchronized each voxel was with the amygdalae during the resting scans of that day.

Change maps in rsFC from the first scanning day to the last were created by first transforming both maps into the high-resolution anatomical space of the subject. Then, they were subtracted (last day minus first day) to yield a seed-connectivity change map. Finally, these seed-connectivity subtraction maps were analyzed at a group level by transforming all participants’ maps to the common MNI space (via a non-linear registration) and adding them. No formal statistics were performed due to the small sample size and the exploratory nature of the study. Thus, an arbitrary threshold was set. The threshold was set such that 5000 voxels survived (in the high-resolution anatomical space) to allow examination of those regions showing the greatest change in connectivity after the NF training. This approach was used to identify peaks in the group map and was also used on the individual subject maps to explore which subjects showed the connectivity changes identified at the group level.

## Results

### Demographics

Participants A, B, and C were adult males of 36, 46, and 30 years of age. All had a similar and moderate exposure to combat (i.e., participants A, B, and C scored, respectively, 22, 24, and 23 on the CES).

Participant’s medical records indicated that participants A, B, and C had a formal diagnosis of PTSD, respectively, for 6, 2, and 3 years when enrolled in this study. Prior to this study, all participants underwent pharmacological and/or psychological treatments. In particular, participant A received 13 weeks of pharmacological treatment and several psychoeducation and mental-health visits at the VA prior to this study. participant B received 13 months of weekly individual psychotherapy prior to the NF intervention at the VA hospital. Participant C received a month-long in-patient care for PTSD followed by a full 12-week course of cognitive processing therapy at the VA hospital. He also received concomitant pharmacotherapy.

During the intervention, participants A and C were on a stable pharmacological dose (for at least 3 months before the intervention started), and participant C was attending individual counseling and group peer-to-peer psychotherapy (initiated more than 3 months before the intervention). At the time of the study, participant B was not receiving any pharmacological nor psychological treatment.

### Behavioral Findings

#### PTSD Symptoms

##### CAPS Scores

Participants A, B, and C had total lifetime PTSD scores of 74, 83, and 88, respectively (i.e., severe-to-extreme symptom range) (70).

**Figure 2:**
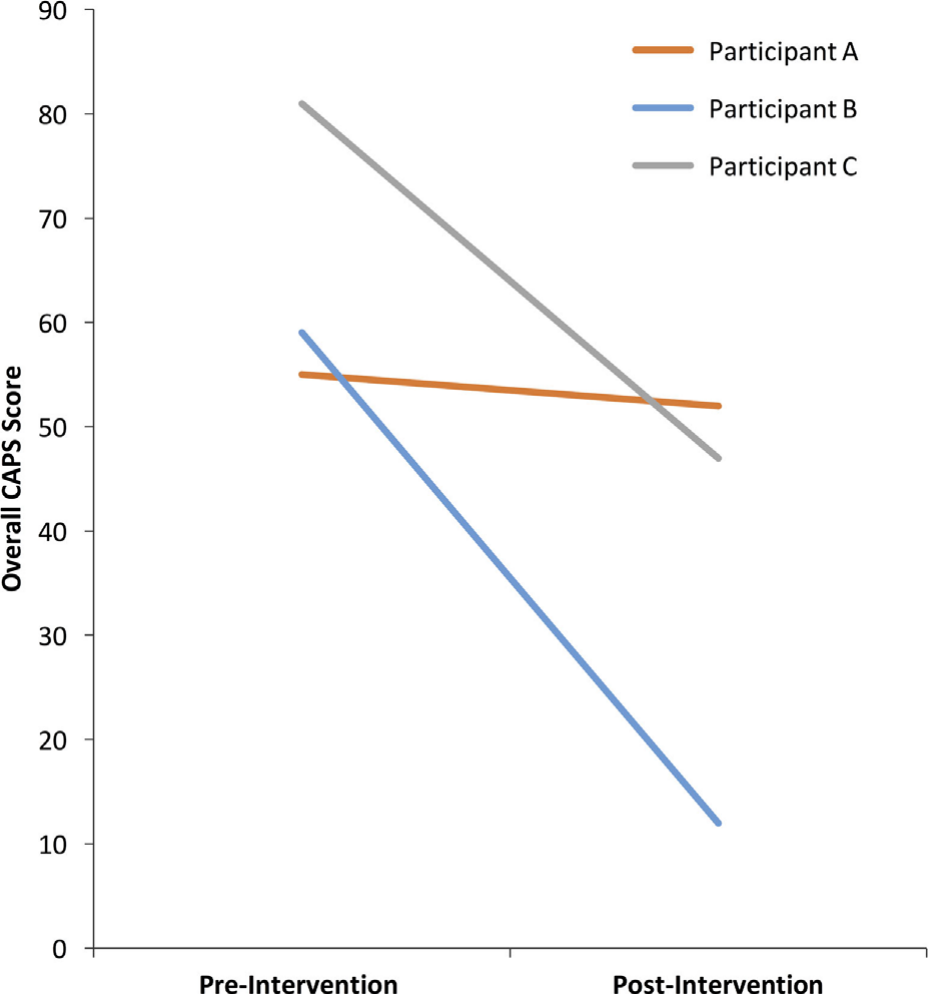
**CAPS scores before and after the NF intervention**.

The past-month CAPS total scores before and after the intervention are shown in Figure 2. According to the CAPS diagnostic categorization (70) before NF training participants A and B with scores of 55 and 59, respectively, belonged in the moderate symptom range (40–59) and participant C, with a score of 81 was in the extreme symptom range (80+).

According to Weathers et al. (70) diagnostic categorization of PTSD symptoms, an asymptomatic clinical presentation is defined as a total score of below 20 on the CAPS. A 10-point decrease in the CAPS total score is considered a reliable marker of clinically significant change (9). Participant B, with drop of 47 points and Participant C with a drop of 34 points had clinically significant improvements in PTSD symptoms (Figure 2). Moreover, participant B achieved asymptomatic presentation with a final CAPS score of 12. Participant A, with a drop of three points did not achieve a clinically meaningful change on the CAPS (Figure 2).

##### PCL-M Scores

**Figure 3:**
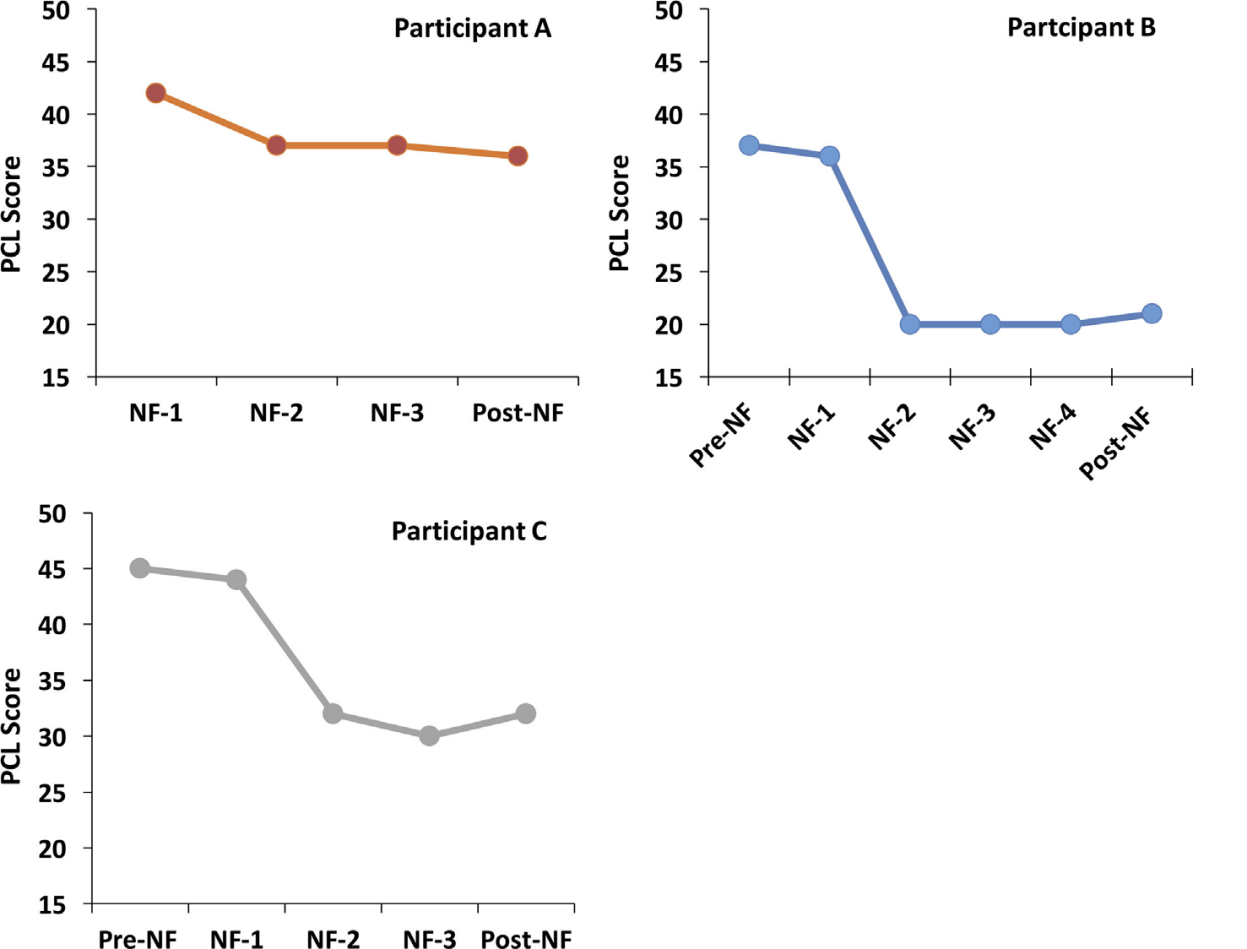
**PCL scores before, during, and after the NF training**. NF-1 represents the PCL-M scores collected just before (but on the same day) as the first NF. Pre-NF represents assessments collected 1 week before the first NF. Post-NF represents data collected 1 week after the last NF session. All NF sessions shown for each subject were scheduled 3–4 days apart.

Post-traumatic stress disorder symptoms were also monitored by the self-administered PCL-M questionnaire in each session. A PCL-M score in 17–33 range is considered to represent low symptomatology, a 33–44 range is considered moderate, and 44–85range is considered high (71). Empirical data suggest that a 5–10 point change is statistically reliable (on an individual basis), and that a 10–20 point change is clinically significant (71). Participant A had a reliable drop in severity score (i.e., five points) from 42 to 37 point, and both participants B and C had a clinically meaningful drop of 16 (from 37 to 21) and 13 points (from 45 to 32), respectively. Interestingly, all participants had the largest drop in severity of PCL-M scores after the first NF session (see Figure 3).

### Neuroimaging Findings

#### Group-Level rsFC Changes post-NF

**Figure 4:**
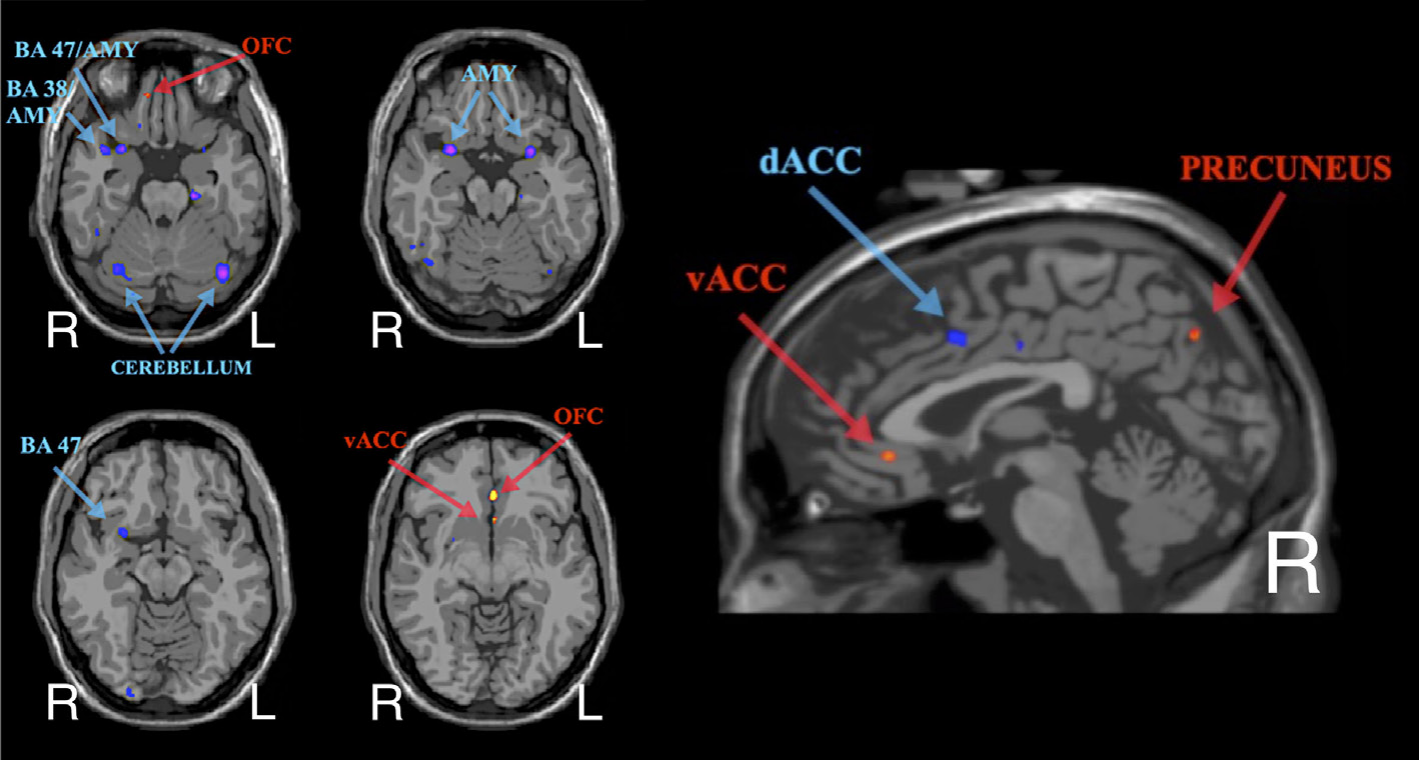
**Group-level average of subtraction seed-connectivity maps (ROI = bilateral amygdalae) showing the peaks in rsFC changes post-neurofeedback (in common anatomical space – i.e., MNI brain)**. Red/Yellow indicates an increase in rsFC, while Blue/Purple indicates a decrease.

As shown in Figure 4, the group-level results from the resting-state seed-connectivity subtraction maps (i.e., last minus first scan) show a pattern of increased rsFC between the amygdalae and regulatory regions in the orbitofrontal cortex (OFC) and the ventral anterior cingulate cortex (vACC). Moreover, a reduction in connectivity between the amygdala and several salience network areas was observed, including the anterior insula, the dorsal anterior cingulate cortex (dACC), and temporal areas surrounding (and including) the amygdalae (for more details, see Table S1 in Supplementary Material).

Notably, the individual subtraction maps were qualitatively similar to the group-level map. Furthermore, none of the group-level findings were contradicted at an individual level (e.g., increased instead of decreased connectivity post-intervention).

## Discussion

This pilot study represents, to the best of our knowledge, the first investigation of the feasibility of rt-fMRI NF in PTSD patients. The main hypotheses were largely supported by our findings. First, all three participants tolerated the NF training well. Second, all patients showed some degree of improvement on the CAPS and PCL-M symptoms scores. Moreover, despite the chronicity of their PTSD symptoms, two participants experienced large (and clinically meaningful) symptom improvements. Third, the changes in rsFC post-treatment were consistent with normalization of PTSD-specific brain patterns across all participants.

### Symptom Improvements

Clinician-Administered PTSD Scale and PCL-M scores provide different windows on symptom severity: the CAPS is a lengthy assessment involving a clinician, while the PCL-M is a brief self-report based measure. Importantly, recent evidence suggests that the PCL may be a more sensitive measure of clinical change than the CAPS in clinical trials (71). However, in this study, the patterns of symptom changes measured are generally consistent for the two measures. Differences in the clinical measures before and after the NF intervention suggest that large symptom improvements occurred for participants B and C who achieved substantial and clinically significant drops in CAPS severity scores, which were mirrored by clinically meaningful changes on the PCL-M. Moreover, post-intervention, with a final CAPS score below 20 points, participant B achieved an asymptomatic presentation. On the other hand, participant A achieved a smaller, yet reliable improvement on the PCL-M, and only a small drop on the CAPS score.

Interestingly, the PCL-M scores revealed that all participants consistently had the largest symptom drop after the first full session of functionally defined NF (NF-1 in Figure 3), which was maintained during the following sessions and post-intervention. This suggests that the symptom improvements observed post-NF training are unlikely to be caused by random symptoms fluctuations over time. Although the largest symptom improvement followed the first NF session, it is unclear whether subsequent sessions may be important for consolidating that learning. Furthermore, as patients were not followed after the final CAPS assessment, it is unclear how long symptom improvements persisted. More research is needed to address these questions.

### Changes in rsFC

Although the changes in rsFC in this study have not been corrected for multiple comparisons and, thus, should not be considered confirmatory, they are worth examining in a qualitative manner as they can inform future hypotheses. The similar patterns of changes in rsFC across participants suggest that the NF may have worked via consistent neurophysiological mechanisms. These changes included (1) increased rsFC between amygdalae and orbitofrontal/ventral anterior cingulate regions (OFC/vACC) and (2) decreased functional connectivity between amygdalae and other parts of the salience network.

Increased rsFC between amygdalae and OFC/vACC is consistent with the traditional neurocircuitry model of PTSD and with current understanding of PTSD pathophysiology (42, 43, 72). In particular, the disruption in the connectivity between regulatory frontal areas (including medial OFC and also the vACC) and the amygdalae is believed to underlie various PTSD symptomatology, such as hyperarousal and alterations in fear extinction processes (43, 72–74). Thus, the preliminary findings from this study suggest that this NF protocol may help to normalize fronto-limbic alterations in PTSD. Interestingly, this finding resonates with neuroimaging studies of other clinical interventions, suggesting that both behavioral and pharmacological interventions also facilitate the normalization of fronto-limbic circuitry in PTSD (75–80).

Decreased connectivity within the salience network is also highly consistent with neurobiological models of PTSD. Abnormally high levels of activity in salience network areas, such as the amygdalae, anterior insulae (AI), and dorsal ACC (dACC) have been linked with severity of PTSD symptoms, with treatment-resistance and also with increased familial vulnerability (42, 43, 81–84). As further evidence of the causal role of the salience network areas in the pathophysiology of PTSD, recent data suggest that positive responses to clinical interventions in PTSD are associated with reduced activity in regions of the salience network (especially the amygdala and dACC) (80). Also the rsFC literature has shown a consistent pattern of abnormally high connectivity within the salience network in PTSD (51, 54, 55). In line with the literature, the rsFC findings from this study revealed (both at an individual and group level) a decrease in rsFC between the amygdalae and other salience network hubs after the NF training. In particular, reduction in the amygdalae’s rsFC were found with (i) the amygdala itself, (ii) with limbic regions bordering the amygdalae, including orbital cortex (BA 47) and the temporal pole areas (BA 38), (iii) with the brain tissues between (and including) the amygdalae and the AI, and also (iv) with the dACC.

One of the difficulties intrinsic to NF training is to target the relevant brain regions or networks (31). Thus, increasing our understanding of the neurological underpinnings of PTSD and the neurophysiological effects of NF training in PTSD can have far-reaching implications for the refinement, and success of rt-fMRI NF.

### Limitations

Despite the encouraging results, this pilot study has several shortcomings. The major limitation is the very small sample size. The goals were to develop a NF protocol for PTSD patients and to test its feasibility and preliminary promise for treating this highly vulnerable psychiatric population. In line with the pilot nature of the research, there were some irregularities. For example, different approaches were used to define the target region. Also, the number of NF sessions received varied across participants. Moreover, the unblinded design does not allow us to control for placebo or social desirability effects on symptoms ratings. The next phase of research should aim to test the current NF protocol in a standardized, randomized study with a larger sample size.

Furthermore, due to the lack of a control group, it is not possible to exclude that the improvements in PTSD symptomatology are due to factors unconnected to the hypothesized mechanisms of action of NF intervention. Other factors may have contributed to the observed symptom reductions, such as (i) exposure to trauma memories [a large body of evidence suggests that simple exposure to trauma imagery can reduce PTSD symptoms (85, 86)], (ii) placebo effects, (iii) social desirability effects, or (iv) random symptom fluctuations across time. However, prior to this study, all three participants had been exposed to psychological treatments and/or mental-health visits, and all had chronic PTSD symptoms. Thus, exposure to traumatic memories and placebo effects are unlikely to explain the symptom improvements experienced by these patients. Furthermore, the temporal pattern of symptom improvements (time-locked to first NF session) (Figure 3) and the changes in rsFC observed (consistent with known neurobiology of PTSD) make the social desirability effect and random symptom fluctuations equally unlikely explanations. In summary, due to the absence of a comparison control group (and the small sample size), no inference can be made about efficacy of the NF intervention for PTSD at this stage. However, the results from this pilot study are promising enough to motivate future investigations into the efficacy of this intervention.

Another limitation is that the patients in this study were not followed clinically after the intervention. Therefore, the persistence of their symptom improvements following NF is unknown. Depending on the persistence of effects, follow-up NF sessions may be needed to maintain improvements. This is an important research direction for future rt-fMRI NF studies.

Finally, this study targeted a very specific subgroup of PTSD patients (i.e., male war veterans with chronic and treatment-resistant PTSD). Thus, it may not be possible to generalize the current findings to other subgroups of PTSD patients [e.g., treatment-naïve individuals, patients with comorbid substance abuse and mood disorders (both very common co-occurring conditions in PTSD), patients with a complex history of trauma, such as sexual abuse and childhood trauma, etc.]. Nevertheless, war veterans are among the group of PTSD patients who are the least responsive to treatment and show the highest drop-out rates (4). Thus, it is likely that the current rt-fMRI NF protocol may be equally (or even more) feasible and potentially effective among other subgroups of adult PTSD patients.

### Conclusion and Final Comments

Unlike other NF methods (such as EEG NF), rt-fMRI NF represents a unique opportunity for targeting specific and deep brain regions involved in the pathophysiology of PTSD. Thus, rt-fMRI NF has the potential to facilitate the translation of neuroscientific knowledge of PTSD into clinical practice. Indeed, the preliminary evidence from this study is encouraging as it suggests that rt-fMRI NF (particularly in a trauma exposure context) on chronic PTSD patients is a feasible and promising new intervention. Further investigation is needed to determine efficacy.

## Author Contributions

All authors (1) made substantial contributions to conception and design, and/or acquisition of data, and/or analysis, and interpretation of data; (2) participated in drafting the article or revising it critically for important intellectual content; and (3) gave final approval of the version to be submitted and any revised version.

## Conflict of Interest Statement

The authors declare that the research was conducted in the absence of any commercial or financial relationships that could be construed as a potential conflict of interest.
