# Supplementary material for: Real-Time fMRI Neurofeedback with War Veterans with Chronic PTSD: A Feasibility Study
Source: Front Psychiatry. 2016 Jun 21;7:111. doi: 10.3389/fpsyt.2016.00111 (PMC4914513; doi:10.3389/fpsyt.2016.00111)
Supplement: Supplementary file 1 [file Presentation_1.PDF]

Supplementary Materials

BDI-II Scores

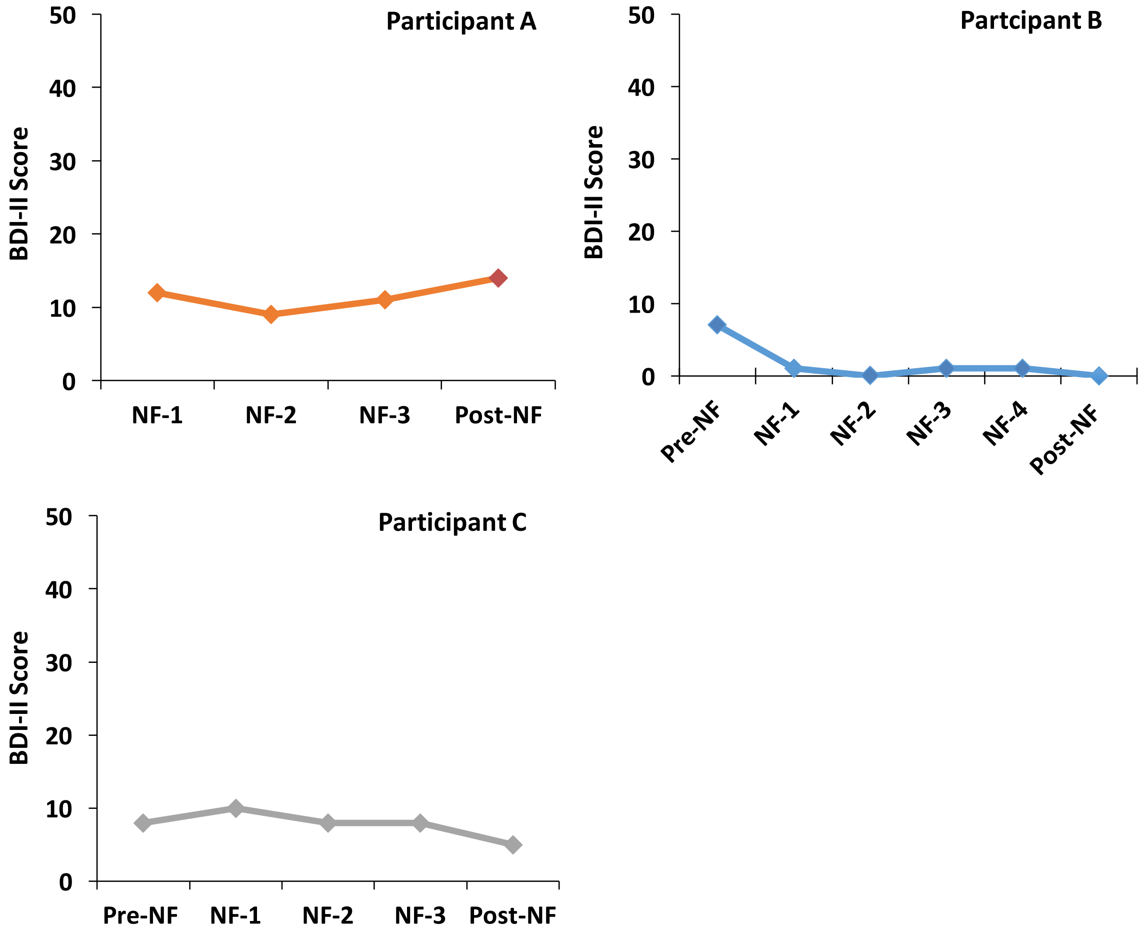

Figure S1. BDI-II scores before, during and after the NF training. NF-1 represents the BDI scores collected just before (but on the same day) as the first NF. Pre-NF represents assessments collected 1 week before the first NF. Post-NF represents data collected 1 week after the last NF session. All NF sessions shown for each subject were scheduled within one week of each other (about 3-4 days apart).

STAI Scores

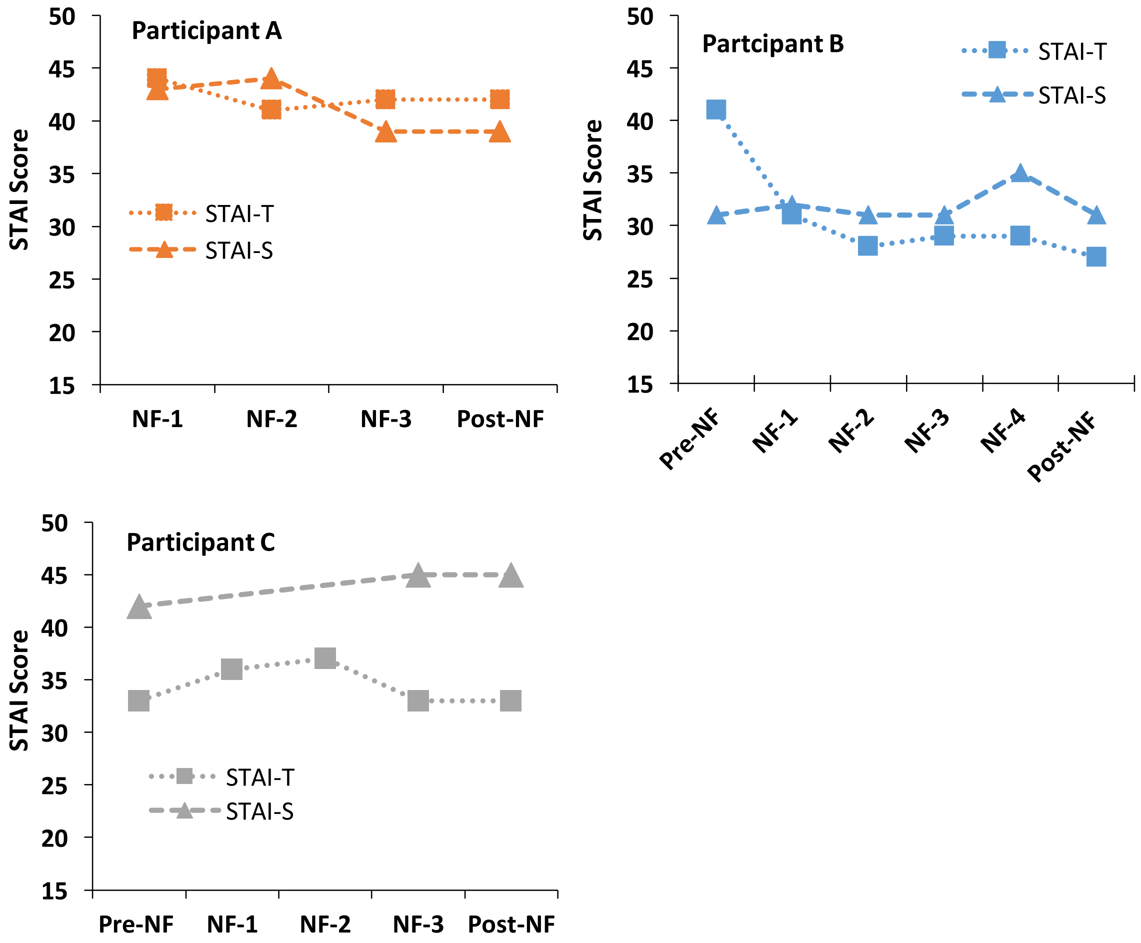

Figure S2. STAI-T (Trait Anxiety) and STAI-S (State Anxiety) scores before, during and after the NF training. NF-1 represents the STAI scores collected just before (but on the same day) as the first NF. Pre-NF represents assessments collected 1 week before the first NF. Post-NF represents data collected 1 week after the last NF session. All NF sessions shown for each subject were scheduled within one week of each other (about 3-4 days apart).

Group Level Changes in rsFC Post-Intervention

Table S1

Group Level Changes in rsFC Post-Intervention – ROI=Bilateral amygdalae

Brain Region	MNI (x, y, z)	Increase/decrease
AMY_L_R	29, 8, -22	↓
BA 47/ AMY_R_L (OFC adjacent to AMY)	-25, 11, -21	↓
BA 38/AMY_R_L (Temporal Pole adjacent to AMY)	40, 7, -23	↓
Anatomical connection AMY-AI_R_L	-28, 9, 19	↓
dACC_R (BA 32)	0, 9, 39	↓
Parahippocampal cortex_L	-19, -20, -23	↓
Cerebellum_R_L	-40, -73, -26	↓
Medial and ventral OFC_R_L (BA 11)	-1,34, -11; 12, 45, -23	↑
vACC_R_L (BA 32, BA 25)	-1, 31, -6; -1, 16, -8	↓
Precuneus_R (BA 7)	6, -79, 48	↓

Note. The ROI (region of interest) was the bilateral amygdalae
Legend. ↓ ↑ = decreased or increased connectivity post-intervention; AMY = amygdala; AI = anterior insula; vACC = ventral anterior cingulate cortex; dACC = dorsal anterior cingulate cortex; OFC = orbitofrontal cortex; BA = Brodmann area.; L = left hemisphere; R = right hemisphere.
